# Hepatic transcriptomic responses in gravid and non-gravid rats exposed to HFPO-DA: Analyses to inform the role of maternal effects in neonatal toxicity

**DOI:** 10.1371/journal.pone.0345643

**Published:** 2026-04-01

**Authors:** Melissa M. Heintz, Chad M. Thompson, Jeffrey C. Wolf, John M. Rogers, Laurie C. Haws

**Affiliations:** 1 ToxStrategies LLC, Asheville, North Carolina, United States of America; 2 ToxStrategies LLC, Katy, Texas, United States of America; 3 Experimental Pathology Laboratories, Inc., Sterling, Virginia, United States of America; 4 ToxStrategies LLC, Raleigh, North Carolina, United States of America; 5 ToxStrategies LLC, Austin, Texas, United States of America; University of South Carolina, UNITED STATES OF AMERICA

## Abstract

Like many per- and polyfluoroalkyl substances (PFAS), toxicity studies for short-chain HFPO-DA (ammonium, 2,3,3,3-tetrafluoro-2-(heptafluoropropoxy)-propanoate) indicate the liver is the primary target of toxicity in rodents. However, neonatal mortality and decreased birth weight have also been reported in rats following oral exposure to HFPO-DA *in utero*. Additional exposure-related effects in neonatal rats included hypoglycemia, decreased liver glycogen, and perturbed hepatic expression of genes related to glucose metabolism and peroxisome proliferator activated receptor (PPAR) signaling. A putative rodent-specific adverse outcome pathway (AOP) network was recently developed using these endpoints and assessed for its applicability to PFAS. AOP 1 in this putative AOP network consists of PPARα activation as one of multiple initiating events, with placental insufficiency, neonatal hepatic glycogen deficit, and hypoglycemia as key events leading to neonatal mortality and lower birth weight. To further inform AOP 1 and investigate whether this altered carbohydrate metabolism liver phenotype observed in rat neonates also occurs in HFPO-DA-exposed gravid and non-gravid adult rats, transcriptomic analysis and glycogen staining were performed on archived female rat liver samples from a 15-d developmental and 90-d subchronic toxicity study. HFPO-DA-mediated changes in hepatic gene expression in adult female rats were consistent with PPARa signaling. Changes in hepatic glycogen content and glucose metabolism-related gene expression did not appear to be dependent on HFPO-DA exposure, suggesting that the altered carbohydrate metabolism phenotype observed in neonatal rat livers is not likely to occur in adult female rats, regardless of pregnancy status. Therefore, key events in this AOP for neonatal mortality and lower birthweight are likely limited to the placenta and/or perinatal rat and are unlikely to be secondary to changes in carbohydrate metabolism in maternal liver. Additionally, findings from this study are consistent with previous mechanistic studies supporting the rodent-specific PPARα mode of action for HFPO-DA-mediated liver effects in rodents.

## Introduction

HFPO-DA (ammonium 2,3,3,3‐tetrafluoro‐2‐(heptafluoropropoxy)‐propanoate, CASRN 62037‐80‐3) is a short‐chain polyfluorinated ether that is one component of the GenX polymer processing aid used in the manufacture of some types of fluorinated polymers. HFPO-DA is part of the broader per- and polyfluoroalkyl substances (PFAS) chemical group, and like other PFAS, several short- and long-term toxicity studies indicate that the liver is the primary target of toxicity in both rats and mice following oral exposure [1,2]. Recent mode of action (MOA) studies for HFPO-DA support that liver effects observed in HFPO-DA-exposed rodents are occurring via the rodent-specific peroxisome proliferator activated receptor alpha (PPARa) MOA [3–9]. Due to key differences in the transcriptional networks controlled by rodent PPARa and human PPARa, it is well-established that only the first key event (KE) of this MOA (i.e., PPARα activation) is shared between humans and rodents, and that the liver effects in rodents associated with the downstream KEs of the PPARa MOA (e.g., hepatocellular hypertrophy and hepatomegaly) are not relevant for human health risk assessment [5,10,11].

**Fig 1:**
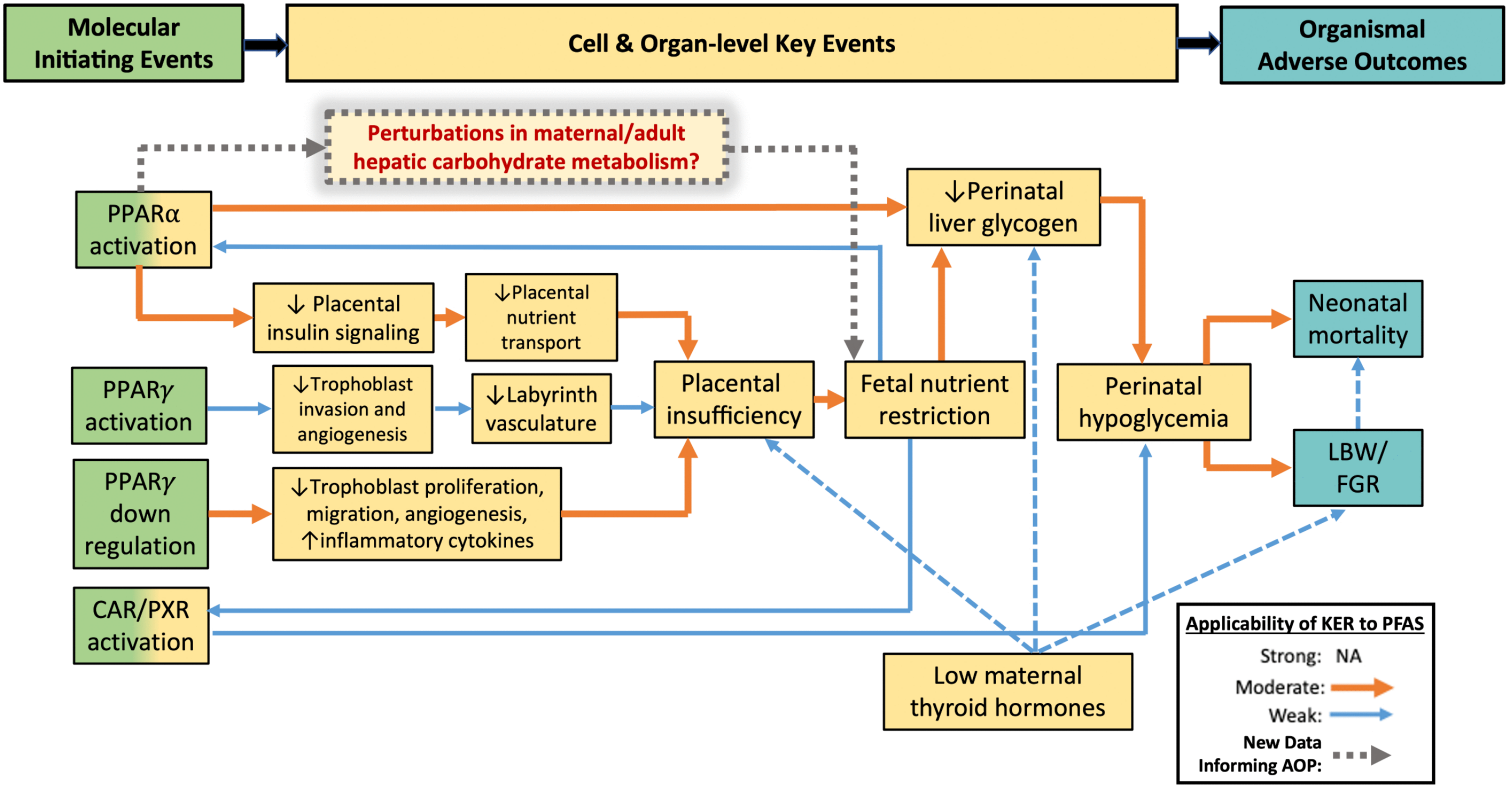
Hypothesized addition (gray dotted lines) to AOP 1 of Rogers et al. [17] AOP network for neonatal mortality and lower birth weight in rodents pending new histopathology and transcriptomic data from the current study. The strength of KERs and applicability to PFAS is indicated by color and thickness of arrows. KER ratings are defined in Rogers et al. [17]. CAR, constitutive androstane receptor; FGR, fetal growth restriction; KER, key event relationship; LBW, lower birth weight; PXR, pregnane X receptor.

In addition to liver toxicity, reproductive and developmental toxicity studies for HFPO-DA have reported exposure-related effects in rodents including changes to maternal gestational weight, placental effects (e.g., increased placental weight and histological placental lesions), decreased fetal/neonatal weights, and reduced neonatal survival [12–16]. Rogers et al. [17] developed a putative adverse outcome pathway (AOP) network for neonatal mortality and lower birth weight in rodents and assessed its applicability to PFAS. AOP 1 in this putative AOP network consists of PPARα activation, PPARγ activation, or PPARγ downregulation as molecular initiating events (MIEs) leading to subsequent KEs including placental insufficiency, fetal nutrient restriction, neonatal hepatic glycogen deficit, and hypoglycemia leading to the adverse outcomes (AOs) of lower birth weight/fetal growth restriction and neonatal mortality in rodents (Fig 1). Importantly, Rogers et al. [17] concluded that while these MIEs and KEs can occur in humans, there are critical species differences (e.g., differences in PPAR structure and function, timeline of liver development) that attenuate the relevance of the key event relationships in this AOP to humans.

A key study supporting the development of AOP 1 in the AOP network proposed by Rogers et al. [17] is a developmental toxicity study by Conley et al. [14] in which Sprague-Dawley rat dams were orally dosed with 10–250 mg/kg-d HFPO-DA from gestation day (GD) 8 to postnatal day (PND) 2 or 1–125 mg/kg-d HFPO-DA from GD 16–20. Decreased birth weight and increased neonatal mortality were observed following *in utero* exposure to HFPO-DA. Other effects reported in Conley et al. [14] included hypoglycemia, increased liver weight and decreased liver glycogen in neonates, as well as alterations to the expression of genes involved in glucose metabolism and PPAR signaling in fetal and neonatal livers using targeted arrays for these pathways. Exposed dams exhibited reduced serum thyroid hormone levels, increased liver weights, and increased hepatic expression of PPAR signaling target genes. Measurement of glucose metabolism gene expression and histopathological evaluation of glycogen content were not performed in maternal rat livers thereby limiting interpretation of whether the changes related to hepatic glucose metabolism were specific to perinatal life stages. Conley et al. [14] suggested a plausible link between reduced neonatal survival and birth weight and the observed alterations in perinatal carbohydrate metabolism.

To determine whether the same hepatic phenotype of altered carbohydrate metabolism observed in *in utero*-exposed rat neonates also occurs in gravid and non-gravid adult female rats following HFPO-DA exposure, whole transcriptome and histopathological analyses were performed on archived adult female rat liver samples from an Organisation for Economic Co-operation and Development (OECD) test guideline (TG) 414 developmental toxicity study (OECD 414) [13] and an OECD TG 408 90-day (subchronic) oral toxicity study (OECD 408) [18], respectively. Findings from these studies can be used to understand the role of HFPO-DA-mediated maternal effects on neonatal toxicity and further inform AOP 1 of the putative AOP network for lower birth weight and neonatal mortality in rodents (Fig 1).

## Materials and methods

### Animal exposure and tissue preparation

The current study utilized archived tissue samples from OECD TG toxicity studies conducted in 2009–2010. Liver samples from these studies were utilized because they originate from well-conducted Good Laboratory Practice (GLP) studies with multiple doses, multiple animals per dose group, and multiple toxicological analyses in the original studies. Moreover, the use of archived tissue samples is consistent with international efforts to limit the use of animals in toxicity testing [19,20]. Although the study designs differ, the same strain of rats and HFPO-DA doses were employed.

The developmental and subchronic toxicity of HFPO-DA were evaluated in GLP OECD 414 and OECD 408 guideline-compliant oral gavage studies, respectively, in Sprague Dawley (Crl:CD(SD)) rats as described in detail elsewhere [13,18]. The technical reports for both toxicity studies are publicly available in USEPA’s Health & Environmental Research Online (HERO) database (HERO IDs: 4222142 and 4222145). Briefly, gravid Sprague Dawley rats were administered deionized water (vehicle control) or HFPO-DA in deionized water at 10, 100 or 1000 mg/kg (n = 22 per dose group) by oral gavage once daily during gestation days (GD) 6–20, for a total of 15 days (developmental study). Non-gravid female Sprague Dawley rats were orally administered the same HFPO-DA doses daily (n = 20 for controls and 1000 mg/kg dose groups, n = 10 for 10 and 100 mg/kg dose groups) for 90 days (subchronic study). Ten animals from each the control and 1000 mg/kg-d groups were used in a recovery study that is not included as part of the present study. Animals were maintained in accordance with the Guide for the Care and Use of Laboratory Animals (National Research Council, 1996) at WIL Research Laboratories, accredited by the Association for Assessment and Accreditation of Laboratory Animal Care (AAALAC) International. All animals were observed twice daily for mortality and moribundity. Clinical examinations were performed daily, and detailed physical examinations were performed weekly. Individual body weights and food consumption were recorded weekly. Animals *in extremis* (e.g., reduced mobility, abnormal body posture or behavior) were euthanized immediately by CO_2_ anesthesia and exsanguination. Additional study details including test substance source and purity, analytical chemistry analyses, animal husbandry, and randomization procedures are provided in the aforementioned technical reports. After the last treatment, rats were euthanized by CO_2_ anesthesia and exsanguination. Livers from adult female rats were fixed in 10% neutral-buffered formalin and embedded in paraffin (FFPE).

### Periodic acid-Schiff staining

For the current study, archived FFPE liver blocks were sectioned approximately 4–6 µm in thickness and mounted to glass slides to be used for Periodic acid-Schiff (PAS) staining or RNA sequencing ca. 2021. For each liver (n = 5 per dose group per study), two serial FFPE sections were mounted and stained with either Periodic acid-Schiff (PAS) or PAS plus diastase (alpha amylase) according to routine methods by Experimental Pathology Laboratories (EPL; Sterling, Virginia). Stained liver sections were evaluated by brightfield microscopy by an American College of Veterinary Pathologists (ACVP) board-certified veterinary pathologist (J.C.W.). The pathologist was unaware of the treatment status of individual animals (i.e., blinded), and scored each liver on a 1–5 scale according to the degree of magenta-colored PAS staining within the cytoplasm of hepatocytes as follows: grade 1 = rare faint staining of individual hepatocytes; grade 2 = occasional areas contain multiple faintly positive hepatocytes; grade 3 = most hepatocytes stain faintly positive; grade 4 = nearly all hepatocytes stain positive, and some stain intensely positive; and grade 5 = nearly all hepatocytes stain positive, and many stain intensely positive.

### RNA preparation and sequencing

For the current study, archived FFPE liver blocks were sectioned from adult female rats from the developmental (15-day) and subchronic (90-day) toxicity studies (n = 5 per dose group per study, for a total of 40 samples). Unstained sections were scraped from the slides and processed according to the TempO-Seq^®^ protocol by BioSpyder Technologies (Carlsbad, CA) to yield libraries of tagged (by sample) and ligated RNA targets, as previously described [21]. Resultant libraries were sequenced using a HiSeq 2500 Ultra-High-Throughput Sequencing System (Illumina, San Diego, CA). TempO-Seq analyses afford the ability to obtain transcriptomic data from FFPE tissues in previously conducted studies thereby limiting the use of animals to conduct *de novo* mechanistic studies. It also has the advantage of obtaining molecular response data near the location where tissue sections were obtained for staining, histopathological scoring, and toxicity evaluation.

### Sequencing data processing and quality assessment

The TempO-Seq® data analysis pipeline was used to analyze raw sequencing data (i.e., FASTQ files) for each sample as previously described [21]. The output of this pipeline was a table containing the number of sequenced reads for each TempO-Seq® probe per sample. Consistent with previous studies, samples were reviewed for inclusion in downstream analyses based on the following sequencing quality criteria: [1] overall sequencing depth ≥2 standard deviations below the mean across all samples, and [2] number of sequenced probes ≥2 standard deviations below the mean across all samples [3,4,6–8]. Samples that did not meet one or both criteria were excluded from subsequent analyses, which were conducted using packages in the R software environment (version 4.4.1; cran.r-project.org/).

### Identification of differentially expressed genes across toxicity studies and dose groups

To account for sample-to-sample variation in sequencing depth, the DESeq2 R package (v1.44.0) [22] was used to normalize count data. Identification of differentially expressed probes (DEPs) and the fold change of these DEPs were determined by conducting statistical comparisons between treatment and control groups of the same study (i.e., developmental or subchronic study) using DESeq2 [22]. DEPs were defined as probes with a false discovery rate (FDR) of <10%, based on *p* values adjusted for multiple testing using the Benjamini and Hochberg (BH) procedure [22]. In the TempO-Seq assay, some (but not all) genes are represented by multiple probes, such that the 22,253 rat probes correspond to 20,922 rat genes. Therefore, differentially expressed genes (DEGs) were identified from the corresponding DEPs (maintaining FDR < 10%).

### Identification of pathway-level responses across toxicity studies and dose groups

Biological pathways associated with transcriptomic responses in the livers of female rats exposed to HFPO-DA were identified by gene set enrichment analysis. For genes (including isoforms of genes) with multiple corresponding TempO-Seq probes, the probe with the highest baseMean (average normalized count value for a probe across all samples) was used in gene set enrichment analysis. Rat gene identifiers were converted into human identifiers (when available) using the R package biomaRt (v2.60.1) [23] based on the Ensembl genome database (http://uswest.ensembl.org/index.html). Although this conversion can introduce assumptions regarding pathway conservation between rats and humans, conversion to human gene identifiers allowed for comparison to previous transcriptomic analyses of livers from HFPO-DA-exposed mice [3,4,8,9] or HFPO-DA-treated primary hepatocytes from mice, rats, or humans [6,7]. Gene expression data were then queried for enrichment of gene sets within the canonical pathway (CP) subcollection (c2.cp.v2024.1) available through the Molecular Signatures Database (MSigDB: http://software.broadinstitute.org/gsea/msigdb/index.jsp); the MSigDB includes gene sets from several pathway databases. The hypergeometric test for overrepresentation was used to identify enrichment of gene sets. DEGs (FDR < 10%) for each dose group within a study were tested for overrepresentation among gene sets in the CP subcollection using the Fisher combined probability test function within the Platform for Integrative Analysis of Omics data (PIANO) R package (v2.20.0) [24]. Gene sets with an FDR < 5% were considered significantly enriched.

### Predicted upstream regulators of differentially expressed genes

QIAGEN Ingenuity Pathway Analysis (IPA, v. 01-22-01; QIAGEN Bioinformatics, Redwood City, CA) software was used to identify predicted upstream regulators associated with DEGs from each study and dose group. Fold change and FDR values (<10%) determined by DESeq2 for DEGs were used to conduct the analyses. For genes with multiple corresponding probes, the probe with the highest baseMean was used.

### Benchmark dose analyses

Dose-response modeling was conducted using the BMDExpress software (v2.3) [25]. Normalized expression data from DESeq2 were loaded into BMDExpress without transformation, using TempO-Seq probe IDs as the gene identifiers. A Williams trend test (with *p* value cutoff < 0.05) was used to identify genes altered by exposure to HFPO-DA. No fold-change filters or corrections for multiple tests were applied. Benchmark dose (BMD) analysis was conducted with the linear, power, hill, 2° and 3° polynomial, and exponential 2–5 models. The models were run assuming constant variance and a benchmark response (BMR) of 1 standard deviation. Significant dose-responsive genes (DRGs) met the following criteria: a best BMD < 10-fold below the lowest tested dose (10 mg/kg-d), a best BMD ≤ the highest tested dose (1000 mg/kg-d), and a winning model fit *p* value ≥ 0.1 [26]. The Reactome gene set collections available within the BMDExpress software were used for functional classification of significant DRGs and calculation of BMDs for enriched gene sets. DRGs with BMD/BMDL >20, BMDU/BMD > 20, or BMDU/BMDL >40 were removed from functional classification analyses. No filters for minimum or maximum number of genes per gene set were applied.

## Results and discussion

### Summary of the toxicological effects observed in female rats from the developmental and subchronic toxicity studies for HFPO-DA

Detailed results of the toxicological endpoints collected during the developmental (15-day) and subchronic (90-day) toxicity studies are provided in the original technical reports [13,18] that are publicly available in USEPA’s HERO database (HERO ID: 4222142 and 4222145). Importantly, these study designs are not directly comparable to one another due to differences in exposure duration and the original purpose of each study (i.e., developmental toxicity versus subchronic toxicity).

Briefly for the developmental toxicity study, one female in the 1000 mg/kg-d group was found dead on GD 20. This female had lower mean body weight gains and food consumption compared to controls during GD 12–18. Liver and kidney changes (moderate coagulative necrosis in the liver and fibrin thrombi in the glomerular capillaries) noted microscopically were considered related to HFPO-DA exposure. Adverse developmental effects from HFPO-DA exposure in the developmental toxicity study were limited to lower mean fetal weights at 100 and 1000 mg/kg-d and early delivery in the 1000 mg/kg-d group. Maternal toxicity (i.e., decreased body weight gain and food consumption) was also observed at 1000 mg/kg-d. Although exposure to HFPO-DA ended approximately two days prior to expected parturition (i.e., GD 22), the lower mean fetal weights at HFPO-DA doses ≥ 100 mg/kg-d suggest that neonatal birth weights would have also been lower in these dose groups. This would be consistent with findings from Conley et al. [14] and the low birthweight AO in the putative AOP network by Rogers et al. [17]. Liver effects reported in adult female rats from this study included increased liver weight following exposure to 100 or 1000 mg/kg-d, focal necrosis at 100 and 1000 mg/kg-d, and hepatocellular hypertrophy at 1000 mg/kg-d (Table 1). These liver effects were generally considered non-adverse by study report authors [12] and are discussed further below.

**Table 1:** Summary of liver effects reported in adult female rats from the developmental and subchronic toxicity studies [13,18].

HFPO-DA mg/kg-d	0	10	100	1000
***Developmental Toxicity Study (15-day)***				
Liver Weight (g)	14.82 ± 1.552	14.93 ± 1.308	16.61 ± 1.946**	19.88 ± 1.689**
Livers Examined	22	22	22	22
Hepatocellular Hypertrophy				
*Minimal*	0	0	0	18
*Mild*	0	0	0	1
Focal necrosis				
*Minimal*	0	0	2	4
*Moderate*	0	0	0	1
***Subchronic Toxicity Study (90-day)***				
Liver Weight (g)	7.63 ± 0.993	7.65 ± 0.629	7.86 ± 1.032	13.53 ± 2.051**
Livers Examined	10	10	10	10
Hepatocellular Hypertrophy				
*Minimal*	0	0	0	10

Note: Liver weights are mean ± standard deviation. ** = *p* < 0.01 relative to controls.

Regarding results of the subchronic toxicity study, one female in the 1000 mg/kg-d group was euthanized on study day 8 *in extremis* due to clinical observations of impaired use of the hindlimbs and forelimbs. On study days 21 and 37, two females in the 1000 mg/kg-d group were found dead; both had similar microscopic findings (renal papillary necrosis, hepatocellular hypertrophy, and lymphoid depletion in multiple tissues) which suggested that these deaths were related to HFPO-DA exposure. The female euthanized *in extremis* died earlier (study day 8) than the other two unscheduled death females (study days 21 and 37) and had macroscopic findings (gross lesions of red areas in the stomach, urinary bladder, and thymus) that were not observed in any other animal from the 1000 mg/kg-d group; thus, the relationship of this early death to HFPO-DA exposure was uncertain. Other adverse effects in surviving female rats included erythrocyte changes associated with regenerative anemia at 1000 mg/kg-d. In addition, liver effects reported in female rats from this study included increased liver weight and hepatocellular hypertrophy at 1000 mg/kg-d (Table 1). Similar to findings in gravid rats from the developmental toxicity study, study report authors and current authors herein considered these liver effects to be non-adverse in the confines of the animal study as they are indicative of PPARa activation [13,18].

The absence of focal necrosis in non-gravid rats in the 90-day study provides some insight into the focal necrosis observed in the gravid rats at 100 and 1000 mg/kg-d in the developmental study. As shown in Table 1, a significant ~2-fold increase in absolute liver weight and hepatocellular hypertrophy were only observed at 1000 mg/kg in non-gravid rats. However, the absolute liver weight was ~ 2-fold greater in control gravid rats compared to control non-gravid rats, and absolute liver weight was significantly increased in the 100 and 1000 mg/kg-d groups of gravid rats, likely because of additional growth from PPARa-mediated hepatocellular hypertrophy (Table 1). Given the absence of focal necrosis in the livers of non-gravid rats exposed to 1000 mg/kg-d for a much longer duration (90 versus 15 days), we hypothesize that the focal necrosis (with minimal severity) in gravid rats was due to excessive liver size as a result of pregnancy and hepatocellular hypertrophy. As such, we do not consider the focal necrosis in the gravid rodents at high doses relevant to humans since PPARa activators have not been found to cause hepatocellular hypertrophy in humans [27].

### Histopathological evaluation of hepatic glycogen content

**Fig 2:**
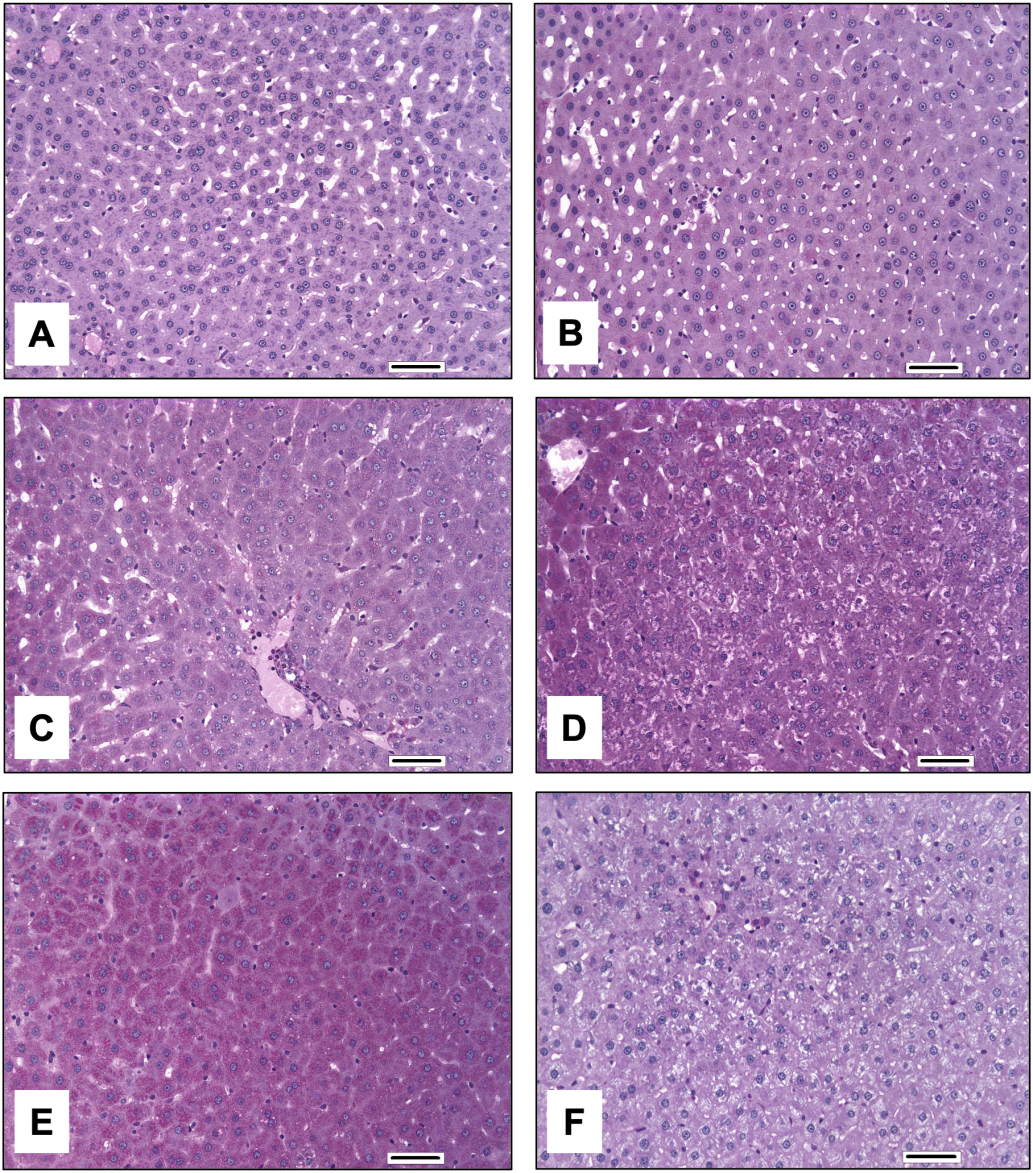
PAS staining of female rat liver sections from the 15-day developmental or 90-day subchronic toxicity studies for HFPO-DA. (A) Grade 1 liver from control, non-gravid female rat from the subchronic study. Minimal PAS staining is evident. (B) Grade 2 liver from 1000 mg/kg-d HFPO-DA-exposed, non-gravid female rat from the subchronic study. Occasional areas exhibit positive cytoplasmic PAS staining. (C) Grade 3 liver from control, gravid rat from the developmental study. Most hepatocytes display faint PAS staining. (D) Grade 4 liver from 100 mg/kg-d HFPO-DA-exposed, gravid rat from the developmental study. Nearly all hepatocytes are positive for PAS, with a few staining intensely positive. (E) Grade 5 liver from 1000 mg/kg-d HFPO-DA-exposed, gravid rat from the developmental study. Nearly all hepatocytes are positive for PAS, and many stain intensely positive. (F) PAS plus diastase staining of the same liver in subpanel 1E (1000 mg/kg-d, developmental study). Essentially all PAS staining is eliminated by diastase, consistent with the presence of glycogen. Note hepatocyte cytoplasmic vacuolation in the absence of glycogen. PAS-positive components in this image include sinusoidal basement membranes and Kupffer cell cytoplasm. Bar = 50 μm.

To investigate whether glycogen depletion occurs in the livers of adult female rats (gravid and non-gravid) exposed to HFPO-DA, liver sections from adult females from the developmental and subchronic toxicity studies were stained with PAS or PAS plus diastase and scored for glycogen content. PAS positivity in hepatocyte cytoplasm was characterized by granular to punctate magenta staining. Exposure to HFPO-DA in gravid or non-gravid female rats did not decrease PAS positivity (Table 2, Fig 2). Diastase treatment eliminated PAS positivity in most liver sections (median PAS score = 0–1), consistent with the interpretation that PAS staining corresponded to the presence of cytoplasmic glycogen. In addition, PAS positivity in the cytoplasm of hepatocytes in livers with PAS scores of 4–5, as observed in both control and HFPO-DA-exposed gravid rats from the developmental study, appeared to be associated with areas of cytoplasmic vacuolation, consistent with glycogen accumulation (see Fig 2F).

**Table 2:** Periodic acid-Schiff (PAS) scores for glycogen content in the livers of gravid and non-gravid female rats from the developmental and subchronic toxicity studies for HFPO-DA, respectively (n = 5 per group).

Study	HFPO-DA Dose (mg/kg-d)	Median PAS Score	PAS + Diastase Median Score
Developmental (Gravid females)	0	5	0
	10	4	0
	100	4	1
	1000	5	0
Subchronic (Non-gravid females)	0	1	0
	10	1	0
	100	1	0
	1000	2	1

### Transcriptomic responses in female rat livers associated with HFPO-DA exposure

During the assessment of sequencing data quality (criteria described in Materials and Methods), it was noted that all samples from the 10 and 100 mg/kg-d groups of the subchronic toxicity study had significantly (adjusted *p*-value < 0.001) lower total number of read counts across all TempO-Seq probes (sequencing depth) compared to samples from other dose groups from the subchronic study (i.e., 0 and 1000 mg/kg-d) and samples from all dose groups from the developmental toxicity study (see S1 Fig; S1A File). Following the removal of these samples, a second assessment of sequencing data quality (according to the criteria described in Materials and Methods) was performed; two additional samples were removed from the analysis, one sample from the 1000 mg/kg-d group of the developmental study and one sample from the 1000 mg/kg-d group of the subchronic study (S1B File). The remaining samples for each dose group (n = 4–5 samples/dose group) included in subsequent transcriptomic analyses exceeded sequencing data quality criteria.

There are several potential reasons for the poor sequencing depth in samples from the 10 and 100 mg/kg-d groups of the subchronic toxicity study. These include issues occurring during the initial sample processing in DuPont [17] (e.g., RNA degradation between necropsy and tissue fixation, weak fixative, unfavorable storage conditions of wet tissues or the tissue blocks) or from issues occurring during the more recent sample processing (e.g., cutting fresh sections, transport from EPL to BioSpyder for RNA preparation or sequencing). However, the exclusion of samples from the 10 and 100 mg/kg-d groups of the subchronic study has minimal impact on the transcriptomic analyses herein because the lowest-observed-adverse-effect-level (LOAEL, defined here as the lowest dose with a statistically significant difference from the controls) for phenotypic histopathological liver lesions was 1000 mg/kg-d in both the subchronic and developmental toxicity studies. Previous meta-analyses indicate that pathway level transcriptional effective doses and apical (e.g., histopathological) effective doses differ less than 2-fold in studies of multiple durations [28]. The results for HFPO-DA in the developmental toxicity study generally follow this pattern (see below). Nevertheless, the exclusion of samples from the low and mid-dose groups of the subchronic toxicity study prevents the ability to evaluate potential dose-response relationships at the transcriptomic level in female rat livers from this study. In addition, there was limited concern regarding RNA degradation in the remaining samples based on detection of common housekeeping genes including *Gapdh*, *Hprt1*, *Tbp*, and *Mrpl13* in control samples from both toxicity studies (see GSE291412).

**Fig 3:**
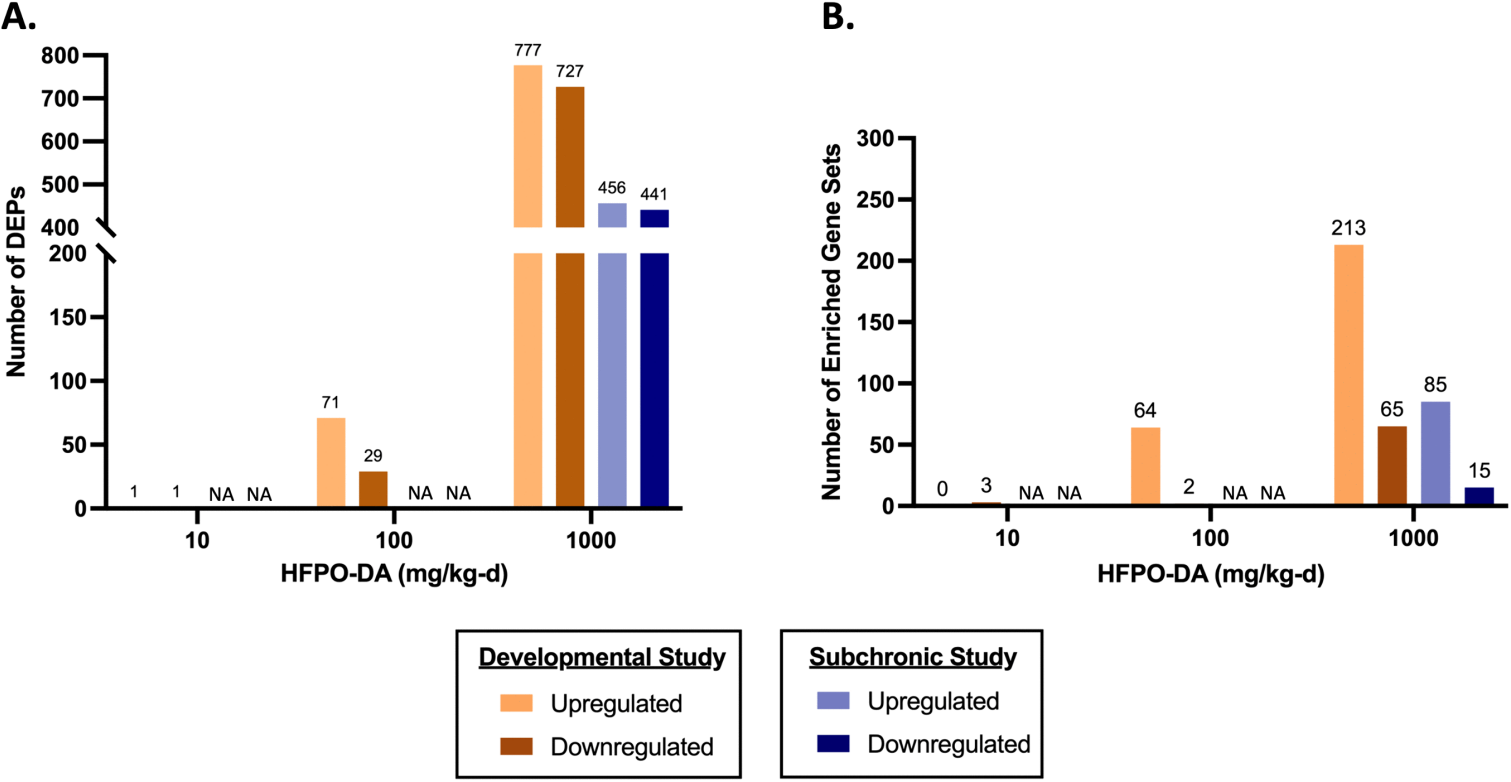
Number of up- and down-regulated differentially expressed probes (DEPs; FDR < 10%) (A) and enriched gene sets (FDR < 5%) (B) in gravid and non-gravid female rat livers from the developmental and subchronic toxicity studies, respectively. “NA” indicates dose groups (i.e., 10 and 100 mg/kg-d) from the subchronic study that were excluded from transcriptomic analyses.

The variance in transcriptomic profiles across all remaining samples from both studies was visualized using PCA (S2 Fig). Based on the separation between samples, study type and dose appear to explain much of the variance observed between samples along PC1 and PC2, respectively. Sample separation patterns observed via PCA were consistent with the number of significant (FDR < 10% and no fold change filter) DEPs across dose groups, with few DEPs in the low and mid-dose groups relative to the control group of the developmental study and a substantially greater number of DEPs in the high-dose groups relative to controls from both studies (Fig 3A; S2 File). Similarly, the number of significant (FDR < 5%) enriched gene sets across dose groups from the developmental study also increased with increasing dose (Fig 3B). The types of enriched gene sets were comparable across toxicity studies, with the topmost significantly enriched gene sets related to PPARa signaling. The topmost significantly enriched gene sets in both studies primarily consisted of an upregulation of fatty acid metabolism and mitochondrial respiration/beta oxidation of fatty acids and a downregulation of complement and coagulation cascades (i.e., innate immune responses) (Table 3; S3 File). Enrichment (which includes both up- and downregulation) of gene sets specifically related to the metabolism of carbohydrates (e.g., glycogenesis, glycogen metabolism, gluconeogenesis) was not observed in dose groups from either study (S3 File). These gene set enrichment results are consistent with previous transcriptomic studies for HFPO-DA in livers from mice [3,4,8,9] and mouse, rat, or human primary hepatocytes [6,7].

**Table 3:** Top 5 most significantly enriched (up- and downregulated) gene sets determined by the hypergeometric test method in female rat livers from developmental and subchronic toxicity studies for HFPO-DA.

Toxicity Study	Upregulated Gene Set Name	Adjusted *p*-value	Downregulated Gene Set Name	Adjusted *p*-value
Developmental	***10 mg/kg-d***			
	–	–	REACTOME Alpha Defensins	8.90E-17
	–	–	REACTOME Defensins	4.86E-12
	–	–	REACTOME Antimicrobial Peptides	1.49E-10
	–	–	–	–
	–	–	–	–
	***100 mg/kg-d***			
	REACTOME Fatty Acid Metabolism	1.87E-22	REACTOME Plasma Lipoprotein Assembly Remodeling & Clearance	0.02185445
	KEGG Fatty Acid Metabolism	8.51E-16	REACTOME Plasma Lipoprotein Remodeling	0.04048488
	REACTOME Mitochondrial Fatty Acid Beta Oxidation	1.14E-12	–	–
	REACTOME Mitochondrial Fatty Acid Beta Oxidation of Unsaturated Fatty Acids	8.73E-12	–	–
	WP Mitochondrial Fatty Acid Oxidation Disorders	9.24E-12	–	–
	***1000 mg/kg-d***			
	REACTOME Fatty Acid Metabolism	5.36E-27	KEGG Complement & Coagulation Cascades	4.36E-18
	REACTOME Aerobic Respiration & Respiratory Electron Transport	7.16E-25	WP Complement & Coagulation Cascades	6.74E-16
	REACTOME Protein Localization	1.54E-17	WP Dengue 2 Interactions w/ Complement & Coagulation Cascades	7.48E-16
	REACTOME Respiratory Electron Transport	1.58E-17	WP Complement Activation	9.45E-15
	WP Electron Transport Chain: OXPHOS System in Mitochondria	1.58E-17	BIOCARTA Complement Pathway	9.44E-11
Subchronic	***1000 mg/kg-d***			
	REACTOME Fatty Acid Metabolism	2.77E-24	KEGG Complement & Coagulation Cascades	9.30E-07
	KEGG Fatty Acid Metabolism	2.04E-16	REACTOME Metal Ion SLC Transporters	0.00275852
	WP Fatty Acid Beta Oxidation	5.01E-11	WP Complement & Coagulation Cascades	0.00275852
	KEGG PPAR Signaling Pathway	8.31E-11	WP Dengue 2 Interactions w/ Complement & Coagulation Cascades	0.00275852
	REACTOME Mitochondrial Fatty Acid Beta Oxidation	1.23E-10	WP Complement System	0.00620001

Key: Dash (-) indicates no significantly enriched gene set(s) (adjusted p value <0.05) for dose group.

**Fig 4:**
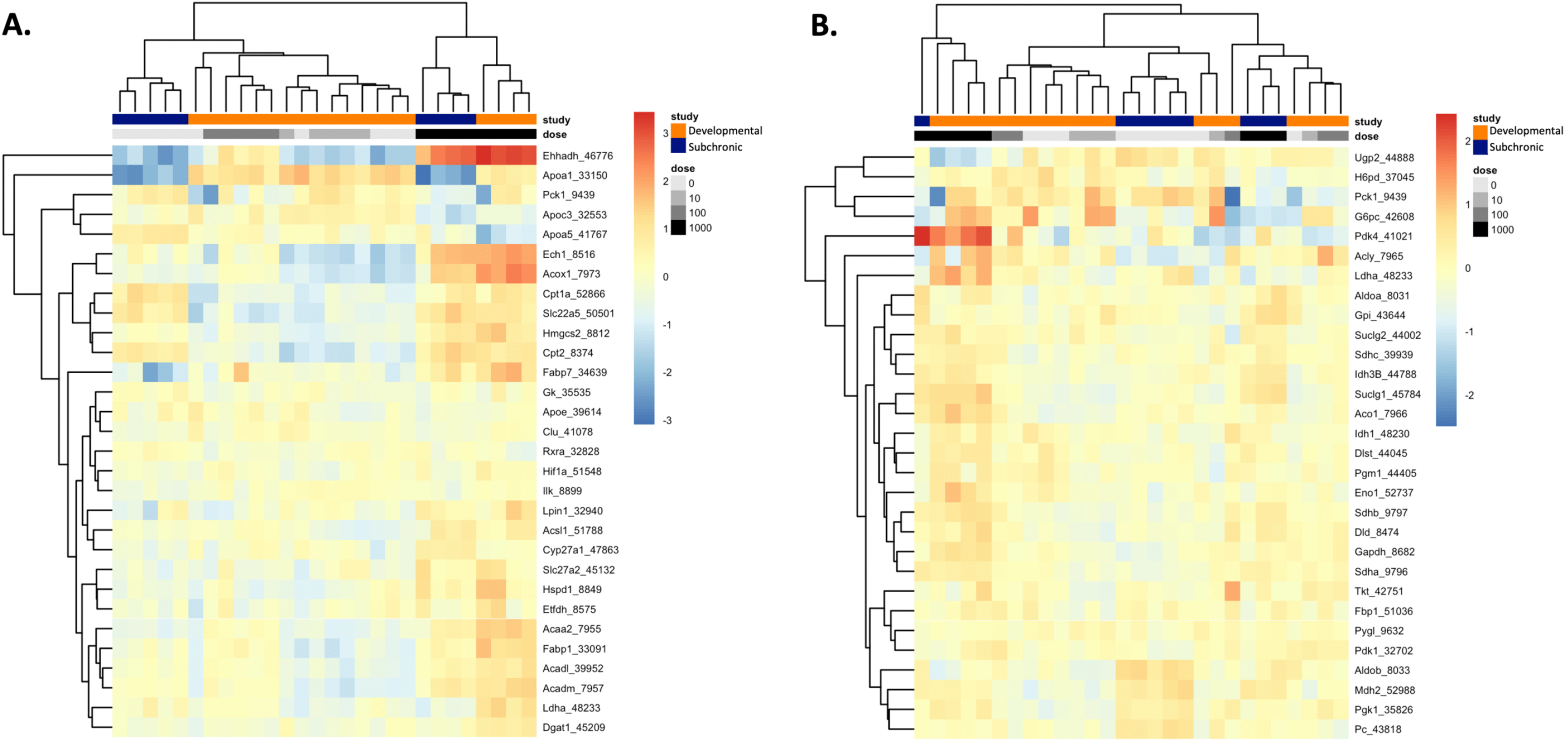
Heatmaps of the normalized relative expression of the top 30 genes (determined by highest base mean) for PPAR signaling (A) and glucose metabolism (B) target genes investigated by Conley et al. [14] across female rat liver samples from the developmental and subchronic toxicity studies for HFPO-DA. Normalized expression level per sample per gene is represented by the distance of each individual sample from the mean expression level for that gene across all samples, with warmer colors (i.e., orange and red) representing an expression level higher than the mean and cooler colors (i.e., yellow-green and blue) representing an expression level lower than the mean. Unsupervised clustering according to Euclidean distances is demonstrated by the ordering of genes (rows) and individual samples (columns), with distances depicted by dendrograms. Color and grayscale identifiers are assigned to each sample (columns) across the top of the heat map by study (orange for developmental, dark blue for subchronic) and concentration of HFPO-DA (grayscale increasing in darkness with increasing dose level).

In addition to the gene sets available through the MSigDB, the expression of specific target genes for PPAR signaling (including genes relevant to PPAR alpha (α), beta/delta (β/δ), and gamma (γ) signaling pathways) and glucose metabolism (including genes involved in the regulation and enzymatic pathways of glucose and glycogen metabolism) investigated by Conley et al. [14] were also examined in female rat livers from the developmental and subchronic toxicity studies herein. Across samples from the developmental and subchronic studies, the clustering pattern of the normalized expression of the top 30 genes (determined by highest base mean) from each target gene list was visualized in heatmaps (Fig 4). Dose level was the primary driver of sample clustering for PPAR signaling target genes (Fig 4A). Consistent with PPAR gene expression changes observed in maternal rat livers from Conley et al. [14], genes specifically involved in peroxisomal or mitochondrial b-oxidation of fatty acids (e.g., *Ehhadh*, *Acox1*, *Ech1, Cpt2*, *Acaa2*, *Acadm, Acadl*) had the greatest magnitude of change in expression. An exception to the dose-driven sample clustering pattern for PPAR target genes was the direction of the relative expression of the PPAR-mediated lipid transport gene, apolipoprotein A-1 (*Apoa1*). Relative *Apoa1* expression was increased across all samples (regardless of dose level) from gravid rat livers (developmental study) and decreased across samples from non-gravid female rat livers (subchronic study) (Fig 4A). We have previously reported dose-dependent decreases in *Apoa1* expression in male and female mice orally exposed to HFPO-DA for 90 days [3]. Previous work by Wu, Sun [29] found that Apoa1 protein infusion in insulin-resistant gravid rats improved insulin sensitivity and reduced inflammation. Altogether, these results may suggest a potential protective role for Apoa1 against pregnancy-induced insulin resistance. In contrast to results for PPAR target genes, the primary driver of sample clustering for glucose metabolism target genes was less clear but did not appear to be driven by HFPO-DA exposure level (Fig 4B). Conley et al. [14] did not evaluate glucose metabolism target genes in maternal rat livers; however, a dose-dependent upregulation or downregulation of certain glucose metabolism target genes was reported by Conley et al. [14] in fetal and neonatal rat livers.

**Fig 5:**
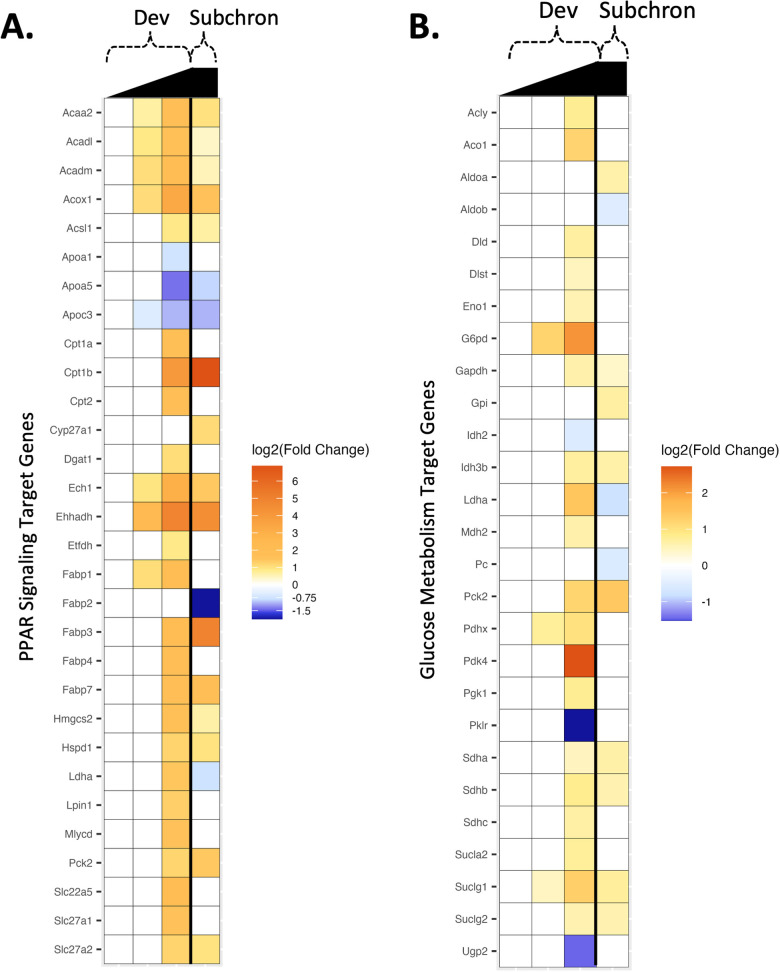
Heatmap of hepatic gene expression (log2(fold change) compared to controls) for PPAR signaling (A) and glucose metabolism (B) target genes in female rat livers from the developmental and subchronic toxicity studies for HFPO-DA (the gene lists are adapted from Conley et al. [14]; see text). Significant (FDR < 10%) DEGs (rows) are indicated by yellow/orange (upregulated) or blue (downregulated) colors; intensity of colors is based on the fold change value for the gene within a dose group and study (columns). White cells indicate that a gene was not significantly altered for a dose group following exposure compared to respective controls. Right triangles at the top of the heatmap indicate increasing dose levels from left to right.

Of the 84 target genes investigated by Conley et al. [14] for PPAR signaling (including α, β/δ, and γ target genes), a total of 30 PPAR signaling genes were significantly differentially expressed across dose groups from the developmental and subchronic toxicity studies (Fig 5A). A greater number of PPAR signaling genes were differentially expressed in the high dose group (1000 mg/kg-d) of the developmental study compared to the subchronic study (i.e., 28 DEGs versus 19 DEGs, respectively). In comparison, a lower number of PPAR signaling genes, 19 total, were differentially expressed in maternal rat livers from Conley et al. [14]. The lower number of genes is likely a result of the lower dose levels (1–125 mg/kg-d HFPO-DA) and shorter exposure duration (5 days) used in Conley et al. [14] compared to the developmental study herein. The types of significant DEGs in maternal rat livers reported by Conley et al. [14] were similar to those measured in female rat livers from the current developmental and subchronic studies and included genes for peroxisomal and mitochondrial β-oxidation (*Acox1*, *Ech1*, *Ehhadh*, *Cpt1b*, *Cpt2*, *Acadl*, *Acadm*, *Acaa2*), ketogenesis (*Hmgcs2*), fatty acid transport (*Fabp1*, *Fabp3*, *Slc27a2*), and mitochondrial protein import (*Hspd1*) (Fig 5A). Many of these genes were also differentially expressed in fetal and neonatal rat livers in Conley et al. [14].

For glucose metabolism, 27 out of the 84 total glucose metabolism target genes evaluated by Conley et al. [14] were significantly differentially expressed across dose groups from the developmental and subchronic toxicity studies (Fig 5B). Similar to PPAR signaling gene expression results, a greater number of glucose metabolism genes were differentially expressed in the high dose group (1000 mg/kg-d) of the developmental study compared to the subchronic study (i.e., 23 DEGs versus 12 DEGs, respectively). However, the overall expression level across DEGs for glucose metabolism was lower compared to expression levels of DEGs for PPAR signaling, with only 3 genes (*G6pd*, *Pdk4*, and *Pklr*) having a log fold change > |1.5|. These three genes code for enzymes critical in gluconeogenesis (*G6pc*, *Pklr*) and acetyl-CoA formation from pyruvate (*Pdk4*) and were only differentially expressed in pregnant rats from the developmental toxicity study (Fig 5B). Although glucose metabolism genes were not assessed by Conley et al. [14] in maternal rat livers, the same genes (*G6pd*, *Pdk4*) or genes with similar functions (to *Pklr*) were also differentially expressed in fetal and neonatal rat livers from Conley et al. [14]. In contrast to the marginal glucose metabolism gene expression changes observed herein, a total of 28 glucose metabolism genes were differentially expressed in neonatal rat livers from Conley et al. [14]. Rat neonates were exposed to lower doses of HFPO-DA (i.e., 10–250 mg/kg-d) *in utero* for a similar exposure duration of approximately 14–15 days (GD 8 - ~ GD 22) compared to the developmental toxicity study assessed herein, suggesting that neonatal rat livers may be more sensitive to glucose metabolism-related gene changes compared to adult female rat livers following HFPO-DA exposure. The greater number of differentially expressed PPAR signaling and glucose metabolism-related genes in adult female rats from the high dose group of the developmental study compared the subchronic study herein may be reflective of the combined impact of chemical exposure in addition to the altered metabolic capacity that occurs normally in rodent livers during gestation [30].

**Fig 6:**
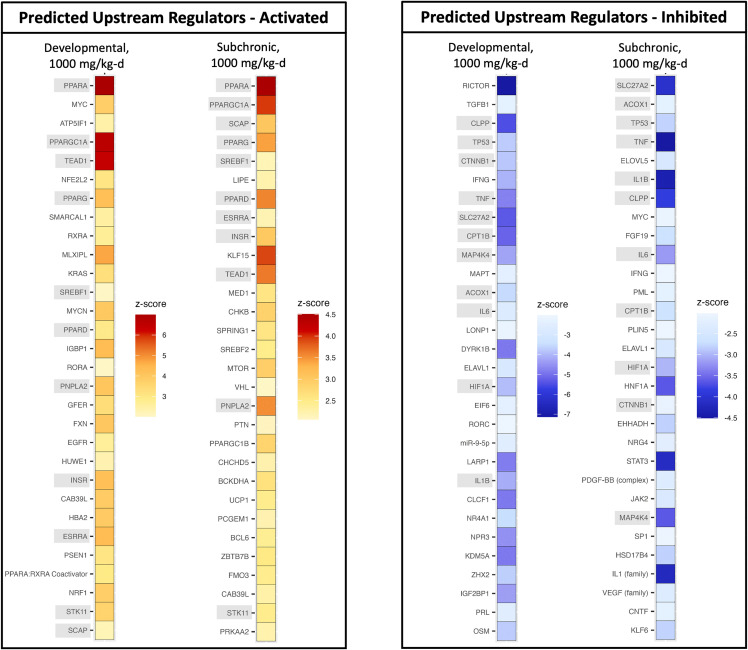
Top 30 predicted upstream regulators (based on *p*-value) for DEGs in female rat livers from the high dose (1000 mg/kg-d) groups of the developmental and subchronic toxicity studies. The top 30 predicted upstream regulators are listed in order of ascending *p*-value. Orange indicates predicted activation, and blue indicates predicted inhibition; the intensity of each color increases with the absolute z-score. Predicted upstream regulators with gray shading indicate upstream regulators that share the same z-score direction and were in the top 30 for both studies.

Despite the differing pregnancy status and exposure duration of female rats from the developmental and subchronic toxicity studies, predicted upstream regulators for the high dose group of the subchronic study had > 60% concordance with predicted upstream regulators for the high dose group of the developmental study (S4 File). Activation/inhibition patterns (z-score) of the top 30 predicted upstream regulators of DEGs (based on *p*-value) for the high dose groups from both studies were visualized via heatmaps (Fig 6). In general, upstream regulators with high absolute z-scores (i.e., > |4.5| z-score) were predicted as upstream regulators of DEGs from both studies. PPARa was predicted as the top activated upstream regulator across the high dose groups from both studies based on both z-score and *p*-value (S4 File).

The top upstream regulators predicted as inhibited included peroxisomal long-chain fatty acid transport enzyme (*Slc27a2*) and rapamycin-insensitive companion of mTOR complex 2 (*Rictor*) (Fig 6). *Slc27a2* was predicted as inhibited for both studies, however *Rictor* was only predicted as inhibited in the developmental study. Rictor is a key subunit of the mTORC2 complex and is involved in the regulation of hepatocellular proliferation and liver regeneration [31]. Gestational hepatomegaly naturally occurs in rodents to meet the increased metabolic demands of pregnancy (see control liver weights in Table 1) [32]; this process in combination with exposure to a PPARa activator such as HFPO-DA, further compounds hepatomegaly (see liver weights in Table 1). Therefore, the predicted inhibition of *Rictor* is likely an adaptive response to moderate the considerable increases in liver size. Likewise, the proto-oncogene *Myc* was predicted as activated in pregnant rat livers and inhibited in non-pregnant female rat livers. This difference in the predicted direction of *Myc* may also be related to the more severe hepatomegaly observed in HFPO-DA-exposed pregnant rats from the developmental study compared to the non-pregnant female rats from the subchronic study. Notably, no predicted upstream regulators for either study type were associated with regulation of glycogen or glucose homeostasis (e.g., insulin receptor [IR], glucagon receptor [GPCR], liver X receptor [LXR], farnesoid X receptor [FXR], carbohydrate response element-binding protein [ChREBP]).

In summary, HFPO-DA-mediated changes in hepatic transcriptomic profiles at the gene and pathway-level in gravid and non-gravid rats were consistent with PPARa signaling. Changes in glucose metabolism-related gene expression in female rat livers were minimal; these transcriptomic findings concur with histopathology results for glycogen content which did not detect exposure-dependent effects (see Fig 2 and Table 2).

### Dose-responsive genes and pathways in gravid rat livers from the developmental toxicity study for HFPO-DA

**Fig 7:**
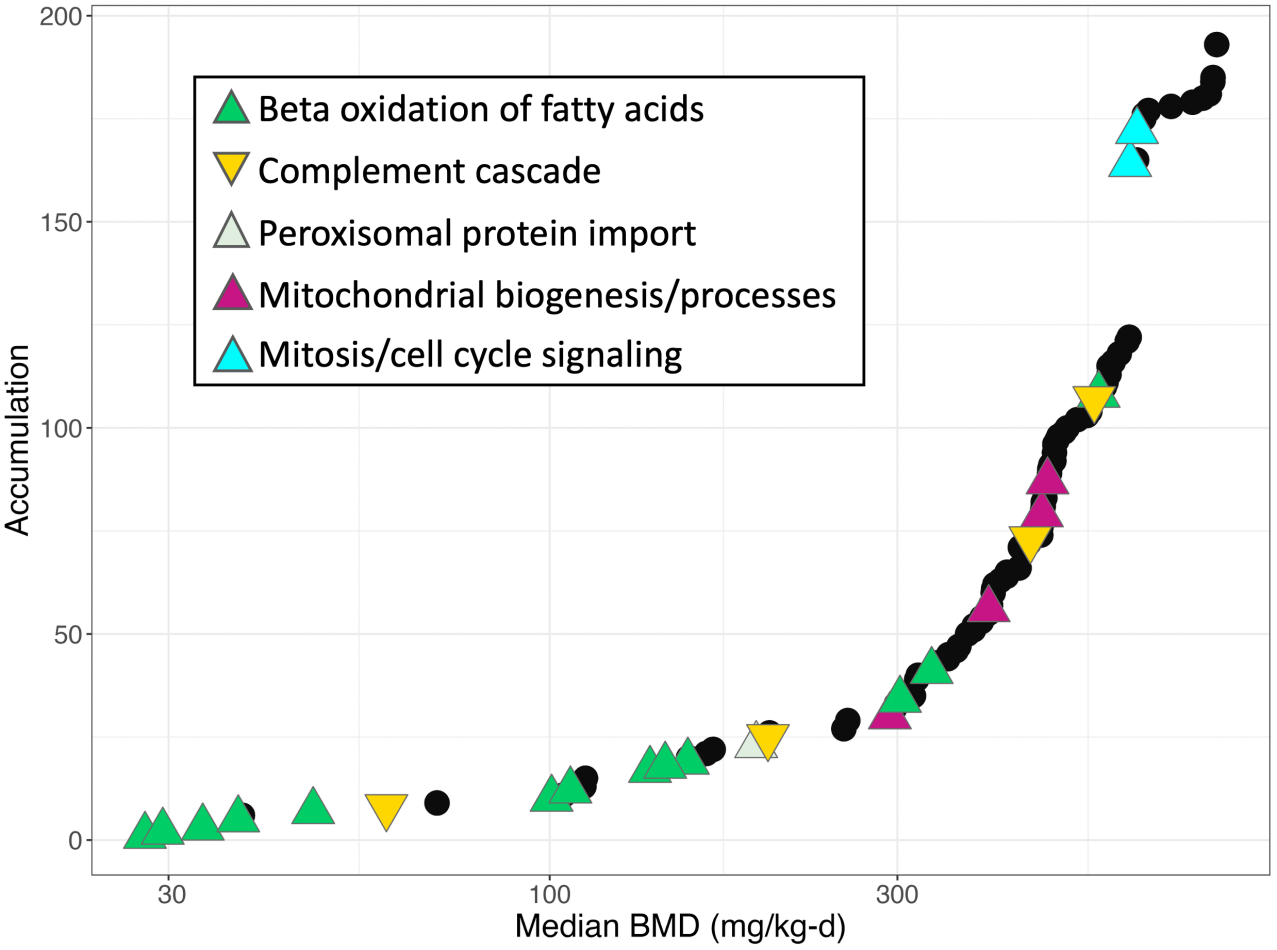
Accumulation plot of median BMDs for significantly enriched pathways (black points; Fisher’s Exact Right *p*-value < 0.05) among DRGs in gravid rat livers from the developmental toxicity study for HFPO-DA. Examples of the types of signaling pathways enriched among DRGs are annotated by color-coded triangles. The direction of the overall transcriptional regulation of the pathway is indicated by the direction of the triangle (pointing upwards = upregulated, pointing downwards = downregulated).

The dose-response of all genes across dose groups in livers from gravid rats in the developmental toxicity study was modeled using BMDExpress v2.3 [25] (S5 File). BMD modeling of transcriptomic responses in female rat livers from the subchronic study was not performed due to the low read counts in the intermediate dose groups (see above). Results from the functional classification (i.e., enrichment of signaling pathways) of significant DRGs in gravid rat livers were comparable to results from the hypergeometric gene set enrichment analyses described in the previous section. Fig 7 shows an accumulation plot of median BMDs of significantly enriched signaling pathways (Fisher’s Exact Right *p*-value < 0.05) in gravid rat livers. Significantly enriched pathways with the lowest median BMDs were related to the upregulation of fatty acid beta oxidation, with median BMDs ranging from 27.85 to ~300 mg/kg-d HFPO-DA (Fig 7). Other upregulated pathways among DRGs in pregnant rat livers were related to peroxisomal protein import (median BMD = 192.01 mg/kg-d), mitochondrial biogenesis (median BMDs = 292.93–482.05 mg/kg-d), and regulation of mitotic cell cycle (median BMDs = 625.63–639.48 mg/kg-d). Substantially fewer pathways were downregulated and were primarily related to complement cascade signaling, with median BMDs ranging from 70.05–456.58 mg/kg-d HFPO-DA (S6 File). Similar to the results of gene set enrichment analysis by dose group, enrichment of signaling pathways related to the metabolism of carbohydrates was not observed among DRGs in gravid rat livers from the developmental toxicity study.

The low-dose transcriptomic enrichment of PPARa signaling pathways, combined with the enrichment of mitotic cell cycle pathways and phenotypic changes (including increased liver weight and incidence of hepatocellular hypertrophy and focal necrosis) at higher doses (i.e., ≥ 100 mg/kg-d HFPO-DA), are consistent with the early KEs of the rodent-specific PPARa MOA [5,10,33]. These results for female rat liver described herein concur with previous mechanistic toxicity studies in mouse liver for HFPO-DA, demonstrating PPARa as the predominant exposure-related transcriptomic signal [3,4,6–8]. Unlike the significant changes in glucose and glycogen metabolism reported by Conley et al. [14] in neonatal rat liver following *in utero* exposure to ≤ 250 mg/kg-d HFPO-DA, there is limited evidence for such changes in the livers of gravid or non-gravid adult female rats exposed to ≤ 1000 mg/kg-d HFPO-DA.

Altogether, these data indicate that the phenotype of altered hepatic carbohydrate metabolism observed in *in utero*-exposed rat neonates is not likely to occur in adult female rats (regardless of pregnancy status) following HFPO-DA exposure. Regarding AOP 1 of the putative AOP network for lower birth weight/fetal growth restriction and neonatal mortality [17], results from the current study indicate that PPARa activation (via HFPO-DA exposure) does not significantly affect hepatic carbohydrate metabolism in maternal/adult female rats, based on both results from histological evaluation of glycogen content and transcriptomic analyses of glucose/glycogen metabolism signaling. Therefore, KEs in AOP 1 are likely to directly affect and/or occur in the perinatal rat and are not secondary to perturbations in carbohydrate metabolism in maternal rat liver as outlined in Fig 1 by gray dotted lines.

## Conclusions

Histopathology and whole transcriptome analyses presented herein demonstrate that the HFPO-DA-mediated phenotype of altered hepatic carbohydrate metabolism observed in perinatal rats is not likely to occur in gravid or non-gravid adult female rats. These conclusions are based on an apparent lack of exposure-dependent changes in hepatic glycogen content using semi-quantitative PAS scoring combined with minimal changes in glucose metabolism-related gene expression using archived FFPE liver tissue from HFPO-DA-exposed gravid or non-gravid adult female rats. The latter finding could be due, in part, to sample age or size, although the sample size used herein (n = 4–5 per dose group) is typical for transcriptomic analyses in animal bioassays including those using TempO-Seq in both fresh and archived rodent tissue samples [3,4,34]. In addition, results from this study provide further support for the rodent-specific PPARa MOA for liver effects in HFPO-DA-exposed rats. An important strength of this study is that the same liver tissues were used for both histopathological examination of glycogen content and transcriptomic analysis, allowing the ability to phenotypically anchor transcriptomic responses. These research findings further inform AOP 1 of the putative AOP network for lower birth weight and neonatal mortality in rodents [17] and suggest that KEs in this AOP are likely limited to the placenta and/or perinatal rat and are not secondary to changes in glucose/glycogen metabolism in the maternal rat liver. While the current study does not address the ability for HFPO-DA to cause such changes in the neonatal liver, species differences in the putative mechanisms of Rogers et al. [17] AOP network, including differences in PPAR structure and function as well as the timeline of liver development, attenuate the relevance of the key event relationships in this AOP to human neonatal outcomes. In addition, based on the results from the current studies herein in gravid and non-gravid adult female rats, perturbations in maternal/adult hepatic carbohydrate metabolism are unlikely to occur in humans following exposure to PPARa activators such as HFPO-DA.

## Supporting information

S1 Fig Initial assessment of sequencing quality of female rat liver samples from the developmental and subchronic toxicity studies. Total number of counts across TempO-Seq probes (i.e., read depth) for each sample. Asterisks (*) indicate dose groups with significantly (adjusted *p-*value < 0.001) lower total number of read counts compared to the other dose groups determined by two-way analysis of variance (ANOVA) F-test followed by a Tukey’s multiple comparisons test to compare the means of each dose group with one another across studies.
(TIFF)

S2 Fig Principal component analysis (PCA) plot of transcriptomic profile variance between samples following removal of samples that did not meet sequencing quality criteria (described in Methods section). Samples removed included all samples from the 100 and 1000 mg/kg-d groups of the subchronic study, one sample from the 1000 mg/kg-d group of the subchronic study, and one sample from the 1000 mg/kg-d group of the developmental study.
(TIFF)

S1 File Sequencing quality across samples. (XLSX)

S2 File Differentially expressed genes from DESeq2 for female rats from the subchronic and developmental toxicity studies. (CSV)

S3 File Gene set enrichment results using the hypergeometric test method. (CSV)

S4 File Predicted upstream regulators (z-score threshold of>|2|) of differentially expressed genes from the developmental and subchronic toxicity studies at 1000 mg/kg-d HFPO-DA. (XLSX)

S5 File BMD modeling of dose-responsive genes in BMDExpress from female rat livers from the developmental toxicity study. (TXT)

S6 File Reactome gene set enrichment of dose-responsive genes in female rat livers from the developmental toxicity study. (TXT)
