# Supplementary figures and images for: Hepatic transcriptomic responses in gravid and non-gravid rats exposed to HFPO-DA: Analyses to inform the role of maternal effects in neonatal toxicity
Source: PLoS One. 2026 Apr 1;21(4):e0345643. doi: 10.1371/journal.pone.0345643 (PMC13042825; doi:10.1371/journal.pone.0345643)

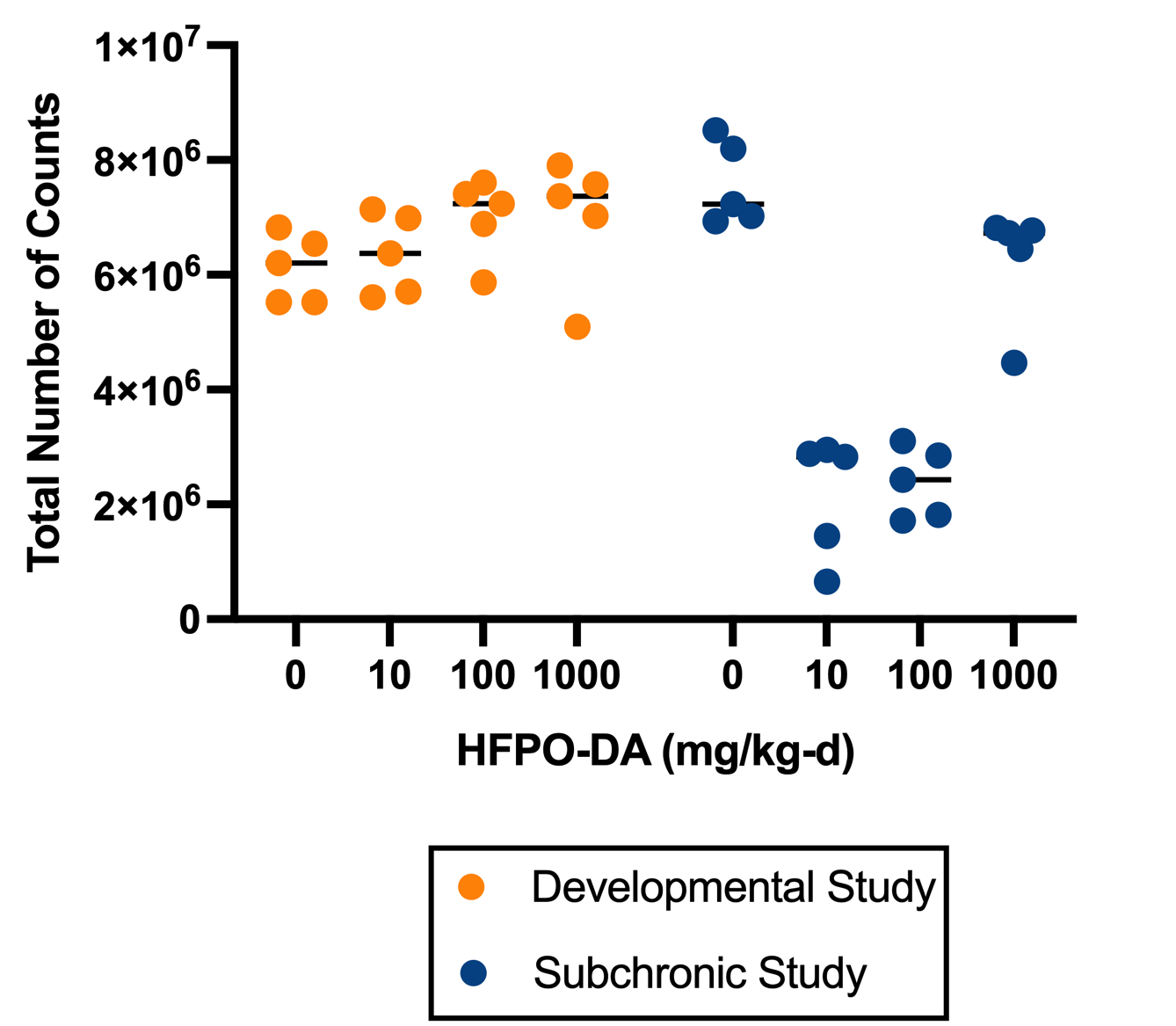

Supplement: S1 Fig — Total number of counts across TempO-Seq probes (i.e., read depth) for each sample. Asterisks (*) indicate dose groups with significantly (adjusted p-value < 0.001) lower total number of read counts compared to the other dose groups determined by two-way analysis of variance (ANOVA) F-test followed by a Tukey’s multiple comparisons test to compare the means of each dose group with one another across studies. (TIFF) [file pone.0345643.s001.tiff]

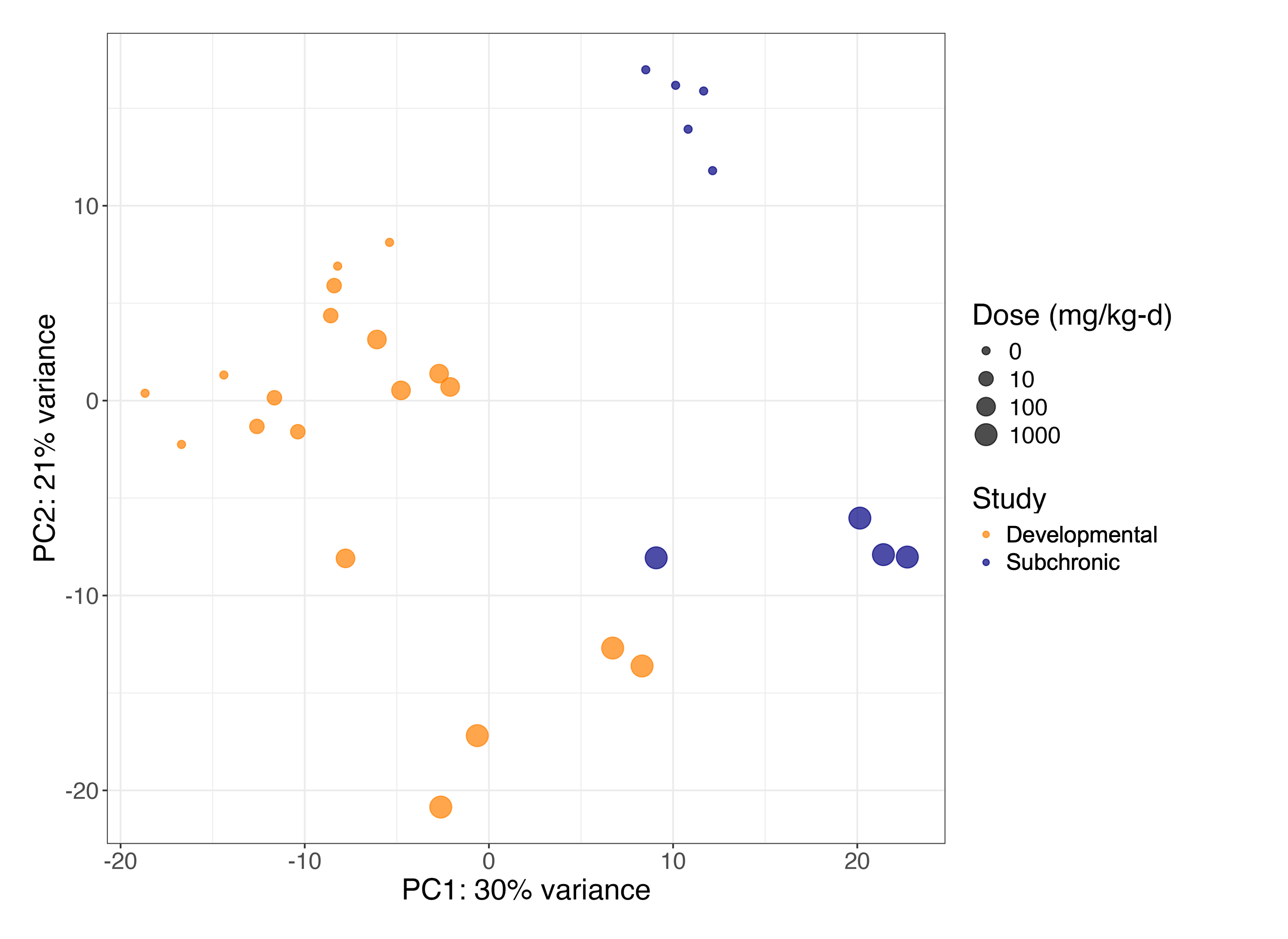

Supplement: S2 Fig — Samples removed included all samples from the 100 and 1000 mg/kg-d groups of the subchronic study, one sample from the 1000 mg/kg-d group of the subchronic study, and one sample from the 1000 mg/kg-d group of the developmental study. (TIFF) [file pone.0345643.s002.tiff]
